# Monitoring of Immune Memory by Phenotypical Lymphocyte Subsets Identikit: An Observational Study in a Blood Donors’ Cohort

**DOI:** 10.3390/jpm14070733

**Published:** 2024-07-07

**Authors:** Marina Di Domenico, Enrica Serretiello, Annafrancesca Smimmo, Fábio França Vieira e Silva, Sonia Anna Raimondi, Caterina Pascariello, Maria Michela Marino, Lorenzo Lo Muzio, Vito Carlo Alberto Caponio, Stefania Cantore, Andrea Ballini

**Affiliations:** 1 Department of Precision Medicine, University of Campania “Luigi Vanvitelli”, 80138 Naples, Italy; marina.didomenico@unicampania.it (M.D.D.); enrica.serretiello@unicampania.it (E.S.); annafrancesca.smimmo@unicampania.it (A.S.); fabio.francavieiraesilva@unicampania.it (F.F.V.e.S.); mariamichela.marino@unicampania.it (M.M.M.); andrea.ballini@unifg.it (A.B.); 2 Clinical Pathology and Microbiology Unit, San Giovanni di Dio and Ruggi D’Aragona University Hospital, 84131 Salerno, Italy; 3 Azienda Ospedaliera “Sant’Anna e San Sebastiano”, 81100 Caserta, Italy; sonia.raimondi@aorncaserta.it (S.A.R.); caterina.pascariello@aorncaserta.it (C.P.); 4 Department of Clinical and Experimental Medicine, University of Foggia, 71100 Foggia, Italy; lorenzo.lomuzio@unifg.it (L.L.M.); vitocarlo.caponio@unifg.it (V.C.A.C.)

**Keywords:** immune system, COVID-19, memory cells’ profile, ABO blood groups, NAÏVE T cells, effector memory T cells, lymphocytes B, lymphocytes T-CD4+, lymphocytes T-CD8, lymphocyte typing

## Abstract

The cross-talk between the innate and adaptive immune response represents the first defense weapon against the threat of pathogens. Substantial evidence has shown a relationship between immune phenotype lymphocytes and COVID-19 disease severity and/or implication in susceptibility to SARS-CoV-2 infection. Recently, belonging to ABO blood groups has been investigated as a correlation factor to COVID-19 disease. This pilot study investigated lymphocyte typing in a cohort of blood donors to understand the underlying mechanism in SARS-CoV-2 infection linked to the blood group. The study cohort consisted of 20–64-year-old subjects, without comorbidities, from both sexes, who were COVID-19 vaccinated with previous or no infection history. Whole blood samples, collected at A.O.R.N. Sant’Anna and San Sebastiano Hospital (Campania Region), were processed by multiparametric cytofluorimetric assay, to characterize CD4+ helper and CD8+ cytotoxic T cell CD3+ subpopulations. The CD45RA, CCR7, CD27, CD28, CD57 and PD-1 markers were investigated to delineate the peripheral T-cell maturation stages. Differences were detected in ABO blood types in CD3+, CD4+ gated on CD3+, CD8+ and CD8+ gated on CD3+ percentage. These results contribute to identifying a memory cell “identikit” profile in COVID-19 disease, thus leading to a useful tool in precision medicine.

## 1. Introduction

The immune system represents a fundamental tool to preserve the organism integrity from external threat. Immunity occurs through two well-recognized mechanisms: innate and adaptive immunity, finely regulated in terms of activation timing and biological factors involved [1]. Innate immunity presents a rapid activation time (from a few minutes to an hour) by the activation of macrophages, Natural Killer cells (NK cells), monocytes and complement system components that activate an inflammatory reaction [2]. In case of eradication failure, pathogens are processed by antigen-presenting cells (APC), able to expose antigenic particles, inducing the specific activation of T and B lymphocytes. The adaptive immunity activation (requires 1–2 weeks) provides cytokine and immunoglobulin release with cellular clonal expansion that generates a long term “memory” in the case of subsequent reinfections [3].

**Figure 1:**
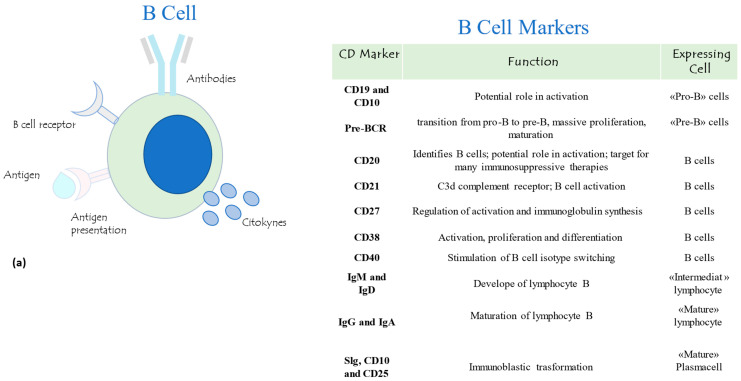

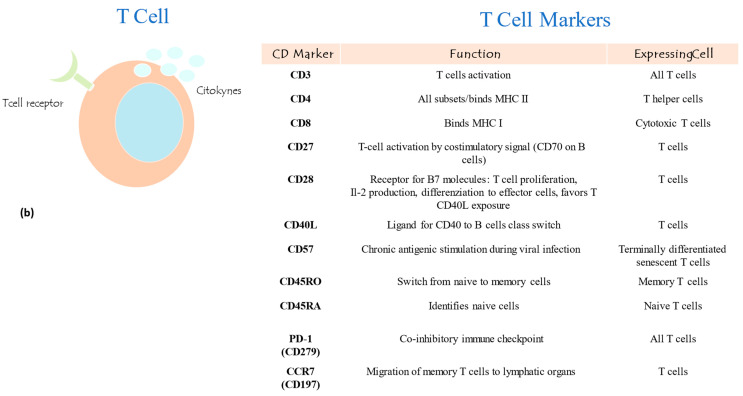
Schematic representation of lymphocyte B (**a**) and T (**b**) markers and their relative functions.

The link between innate and adaptive cell types during infection is finely regulated to perform an efficient immune response. In particular, the B lymphocytes carry out the task of APC cells, directly recognizing the antigen on host membrane cells, that proliferate and transform into the effector cells plasma cells, able to produce antibodies against the specific pathogen. The B cells differ from other lymphocytes by the presence of the B-cell receptor (BCR) [4]. On the other hand, the T lymphocyte TCR/CD4-CD8 receptors recognize the antigenic peptide presented by major histocompatibility complex (MHC) proteins. The main lymphocytes T and B surface biomarkers and their relative functions are reported in Figure 1a,b.

According to their functions, T cells are divided into populations and subpopulations. For the effector T lymphocytes, we find T helper (TH) or CD4+ (subpopulation TH1, TH2, TH3, TH17, TH9, e TFH), T cytotoxic (TC) or CD8+ (Tc double negative and double positive) and T regulator/suppressor TREG (subpopulations: FOXP3+ TREG e FOXP3− TREG). The memory T-cells’ group is made up by the “Naïve” (TN), “central” memory (TCM), “effector” memory (TEM) and “terminally differentiated” effector memory (TEMRA) cells population. In the Natural Killer (NTK) family, we find NKT1, NKT2 and NKT-like. The T lymphocytes γδ are Vγ9/Vδ2 and no-Vδ2+ [5,6,7,8,9,10,11]. T-cell memory subpopulation members differ from each other by the expression of specific markers. Naïve T cells are so defined because they are not already exposed to the antigen. The TN-antigen (MCH-APC) recognition induces the TN activation and proliferation of specific antigen clone cells and differentiation into memory and effector cells [12]. TEM subpopulations migrate to peripheral zone-producing cytokines. TCM can be differentiated in the TEM constituting the reserve for the effector cell population. TEMRAs are characterized by low replicative activity but a high cytotoxicity power [13]. CD28+CD45RA− or CD27+CD45RA− are typical phenotypes of memory T cells, instead, CD28−CD45RA− or CD27−CD45RA− represent memory/effector T-cell phenotype. CD27−CD28− subsets of CD45RA−CD8+ T cells represent high cytokine-producing cells. On the other hand, the co-receptor CCR7 expression in CD45RA−CD8+ and CD27+CD28+ phenotypes did not result in CD27−CD45RA+CD8+ T cells, suggesting its preferentially expression only on naive and memory CD8+ T cells [14]. PD-1 (Programmed Death) represents a checkpoint inhibitor of T-lymphocyte activation, a key biomarker of lymphocyte depletion.

**Figure 2:**
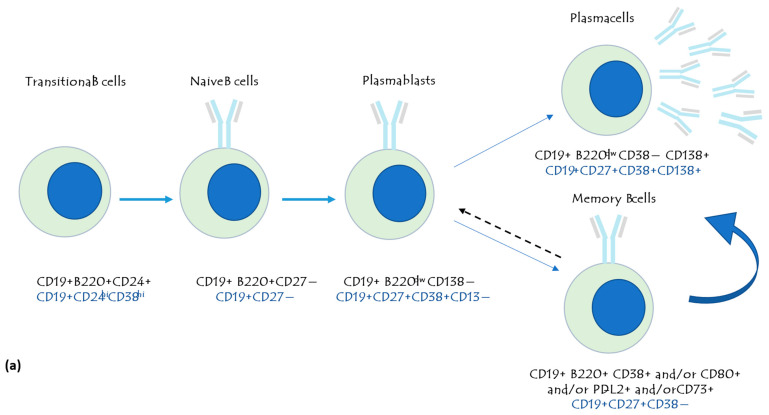

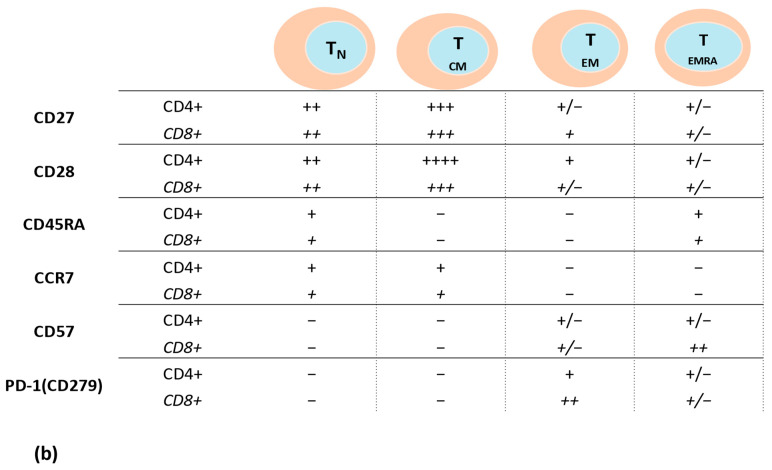
Activation of lymphocyte B-cell (**a**) and peripheral T-cell (**b**) maturation stages and the related surface markers that are expressed.

The exhausted cells expressing PD-1 present decreased proliferation and differentiation, and low cytotoxic activity, and are thus associated with poor control of infections [15]. Exhaustion seems to be a reversible process, restoring an activity power to CD8+ lymphocytes, and conversely to the senescence, a shift towards less functional T cells [16]. CD57 is a marker of terminal differentiation, a signature of the mature NK cells, and its expression increases with aging and during chronic infections [17]. The classification of the different lymphocyte subpopulations, by the expression of peculiar surface markers, is reported in Figure 2a,b.

The association between ABO blood groups and SARS-CoV-2 infection has been poorly investigated. It has been proposed that anti-A and/or anti-B antibodies are able to bind A and/or B antigens expressed on the viral envelope, interfering with virus entry and dissemination [18,19,20,21,22,23]. The increase in ACE1 activity in group A subjects could predispose them to cardiovascular complications, such as the high level of Willebrand factor and Factor VIII levels in ABO type which could contribute to the development of thromboembolic disease and hemorrhage [24,25,26,27,28,29,30,31]. Other literature promotes an association between blood group A and COVID-19 infection [32,33,34,35]. This susceptibility trend was reported by several authors, even if the impact of blood type on clinical outcomes remains unclear.

This pilot study aimed to investigate a lymphocyte “identikit” profile for COVID-19 disease, in a cohort of patients selected from blood groups (A-B-0), by a multiparametric cytofluorimetry approach, to evaluate a possible statistically significant correlation.

## 2. Materials and Methods

### 2.1. Samples Collection

Blood donors were recruited between June and October 2023 in the A.O.R.N. Sant’Anna and San Sebastiano, Caserta Hospital (Italy). The cohort study included healthy blood donor subjects aged 20–64 (median age 46 years old), of both sexes, with or without previous COVID-19 infection, with at least one SARS-CoV-2 dose vaccine administered. Peripheric whole blood samples were collected in a blood collection tube containing anticoagulant and immediately processed in the “U.O.C. Immunoematologia e Centro Trasfusionale” laboratory, to perform multiparametric flow cytometry analysis using DURAClone IM B Cell and DURAClone IM T Cell (BECKMAN COULTER) according to the manufacturers’ protocols [36,37]. The present study was conducted in accordance with the Declaration of Helsinki after obtaining the approval by the Azienda Opedaliero–Universitaria Luigi Vanvitelli–AORN Ospedale dei Colli–Local Ethic Committees: Prot. N 0013965i/(11 May 2023) “Analisi quantitativa e qualitativa delle Vescicole Extracellulari in soggetti ospedalizzati con infezione da SARS-CoV-2″; prot.: ESOCOV”. Cytofluorimetric analysis was performed by the NAVIOS EX 10-color flow cytometer (BECKMAN COULTER) specifically for the clinical laboratory analysis [38]. The data obtained were analyzed by KALUZA C software Ver 2.2 (BECKMAN COULTER) [39].

### 2.2. Inclusion/Exclusion Criteria

Results showing a negative serological analysis for blood-transmissible infections (hepatitis B and C, HTLV, HIV, syphilis) in the enrolled subjects, meant exemption from any pathology or treatment able to interfere with leukocyte parameters. Hence, subjects with any comorbidities, those who were immunocompromised, had a history of cancer or of autoimmune disease, or were undergoing a course of systemic infection, immunomodulating or immunosuppressive therapy, had a presence of a proven severe allergy or had been administered a vaccine less than 3 months before were excluded.

### 2.3. B and T Lymphocyte Subpopulation Panel

The research approach was articulated by a developed roadmap based on outlining the epidemiological context of the infection in the Campania Region, the recruitment of blood donors and the analysis of memory and senescence cells. Both the B and T lymphocyte subpopulation panels which were analyzed were reported in Table 1. Identification of the lymphocyte subset to predict a personalized immune “identikit” profile in COVID-19 subjects was collocated in the early diagnosis, prognosis and predictive biomarker fields as a translational tool.

### 2.4. Statistical Analysis

Descriptive statistics were computed both on the overall population and subgroups, A, B and 0 blood types. In depth qualitative data were expressed as absolute and relative percentage frequencies, whilst quantitative variables were expressed as median and interquartile ranges (IQR) or mean and standard deviation (SD), according to data distribution. Difference between three blood types were assessed by the ANOVA test or Kruskal–Wallis test [40,41,42], whilst the Chi-square test was applied as for qualitative data [43]. When statistical difference was detected, the pairwise *t*-test or non-parametric Mann–Whitney U test with Benjamini–Hochberg correction for multiple testing was performed. Assessment of non-normal distribution was performed by the Shapiro–Wilk test [44], whereas Levene’s test was used to test the equality of variances between blood type subgroups [45]. A *p*-value < 0.05 was considered statistically significant. All analyses were performed by R Statistical Software (v.4.3.1, R Core Team 2023), from an experienced author (A.S.).

## 3. Results

### 3.1. Enrolled Population

Seventy-five blood donors were recruited for this study, but only seventy-two met all the inclusion criteria chosen. Therefore, our enrolled population, 72 SARS-CoV-2 vaccinated blood donors with a median age of 46.0 (20.0–64.0), consists of 23 (31.9%) women and 49 (68.1%) men; mostly residents in Caserta (88.9%). Twenty-one donors possess A blood type (29.2%), twenty-two present B blood type (30.6%) and twenty-nine for O blood type (40.3%). Our population mostly has a positive Rhesus factor (91.7%) (Table 2). The distribution of Rhesus factor by blood type is reported in Appendix, Table A1. Fifty-one donors (70.8%) declared that they had a previous SARS-CoV-2 infection and only three donors (4.2%) declared two previous infections. Mostly of the enrolled population received three doses of the COVID-19 vaccine (80.6%) (Table 2).

**Figure 3:**
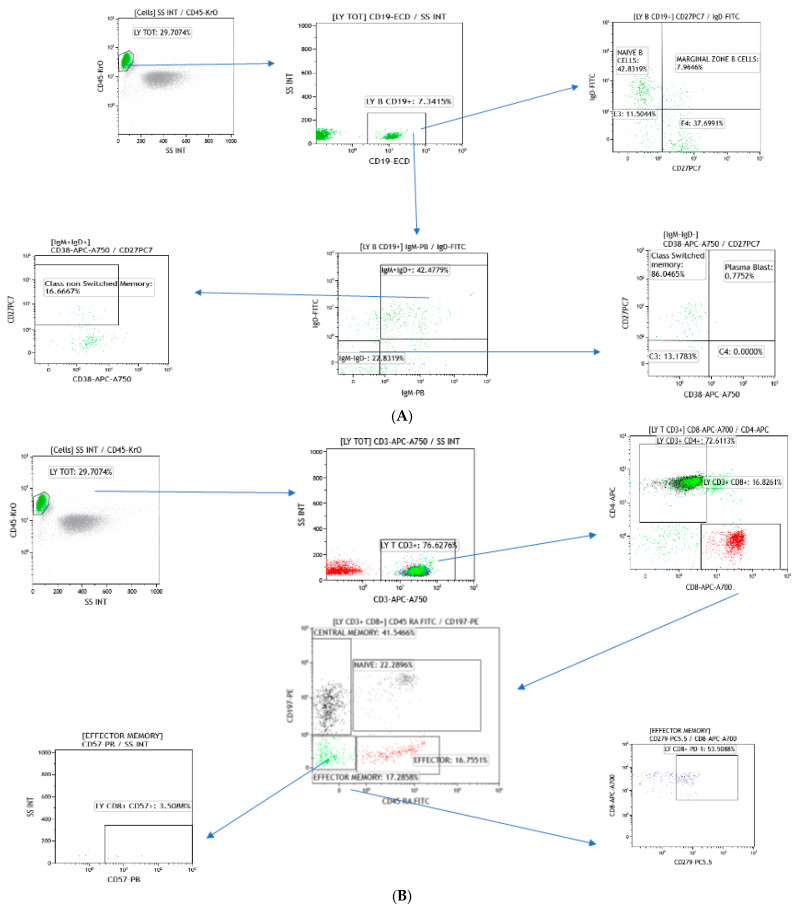
Gating strategy for the identification of the indicated B-cell subsets (**A**) and indicated T-cell subsets (**B**) in healthy donors.

The endpoint of this pilot study was to investigate the difference in immunological profile in the A, B and O blood type using specific lymphocyte subpopulations. Panels of investigated markers, obtained through cytofluorimetric analysis, are presented in Figure 3A,B.

### 3.2. Immuno-Profile Identikit by Blood Type

As already reported, many studies showed a relation between blood type and level of COVID-19 severity or predisposition to the infection. To investigate the underlying variations in this process, we analyzed the immune profile memory related of this cohort of blood donors. First of all, we observed that mean age is quite higher in the B subgroup (47.3 y.o.). Other demographic information was reported in Table 3. Among the three blood type subgroups, the A resulted to be more prone to infection than the others two, since the A blood type has the highest percentage of previous infected donors. Furthermore, donors declaring to have two infections belong to above-mentioned blood type (A). Highest percentage of vaccine’s doses administered (three and four doses) belong to B subgroup (90.1%), followed by the A subgroup (89.7%) (Table 3). Starting with levels of white blood cells, expressed as absolute count and percentage, we detected no statistical differences among the three subgroup (Table 3).

Focusing on lymphocytes, we analyzed the selected lymphocytes B, T-CD4 and T-CD8 subpopulations. Concerning lymphocytes B, although some subpopulations resulted in being higher in one or two subgroups (i.e., Naïve B cells: IgD+ CD27− gated on CD19, B cells IgM+ IgD+ gated on CD19+, CD38− CD27+ gated on IgM− IgD− (switched memory B cells), this was not confirmed by statistical test (Table 4).

Investigating the CD3 lymphocyte T subpopulation, we observed a statistically significant difference among the three subgroups (*p* = 0.026). Using the pairwise test with multiple test correction, we can conclude that higher percentage of CD3 subpopulation in O blood type is statistically different from the percentage in A blood type (*p* = 0.043), whilst the comparison between B and O blood types showed a certain trend toward significance (*p* = 0.078). Furthermore, the CD4 subpopulation gated on CD3 resulted in being statistically different among three blood types (*p* = 0.044). In particular, the comparison of B versus O blood type is statistically significant, meaning that the percentage of CD4 subpopulation gated on CD3 in B blood type is higher than the same subpopulation in O blood type (*p* = 0.047). Concerning lymphocyte T-CD8 subpopulations, we observed a significant difference for CD8 and CD8 gated on CD3 subpopulations (*p* < 0.001; *p* = 0.029). Pairs of blood type resulted in being different for a percentage of CD8 subpopulation are both A versus 0 and B versus 0 (*p* = 0.002; *p* = 008). Therefore, the CD8’s percentage is higher and significantly different from the other two blood types. CD8 gated on CD3 resulted in being significantly different for just B versus O blood type (*p* = 0.031) (Table 5).

**Figure A1:**
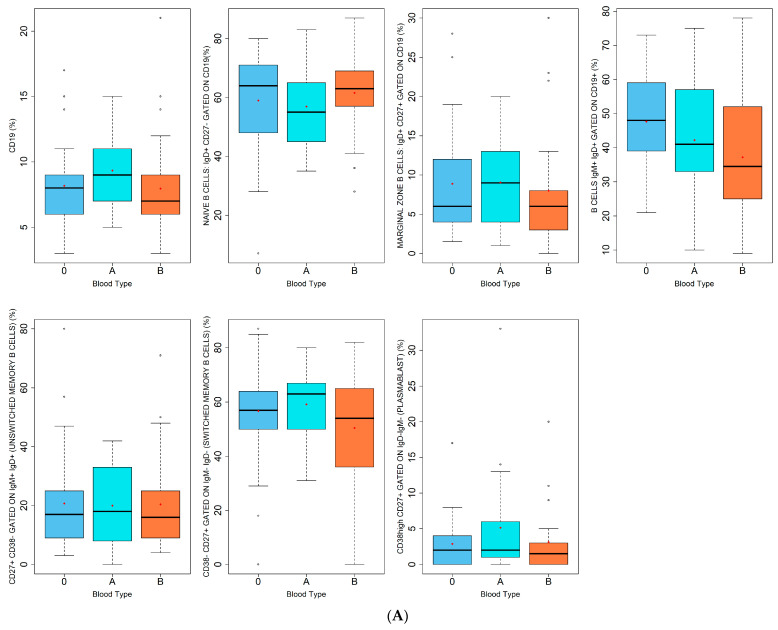

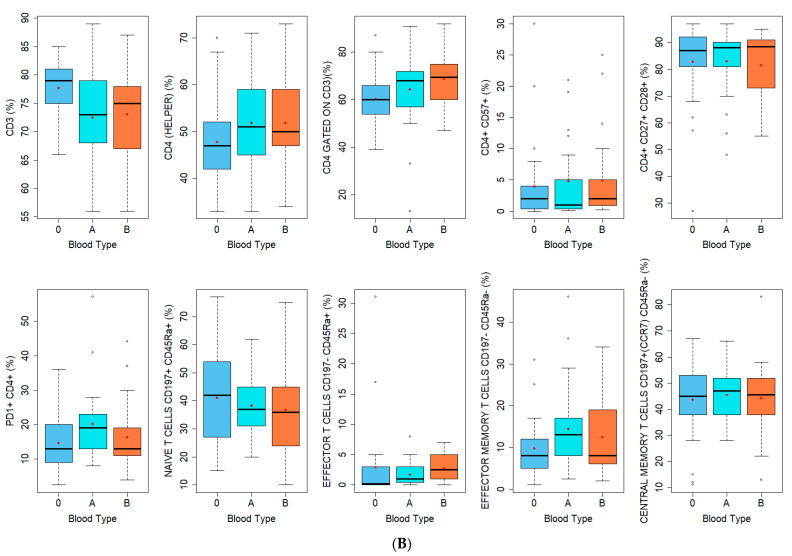

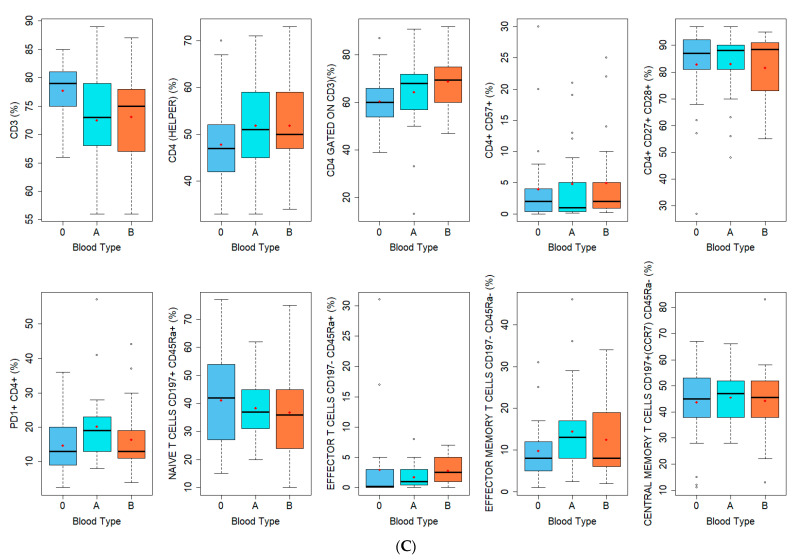
These three boxplots display the observed frequency distribution of all lymphocytes’ subpopulations analyzed, stratified on blood type. Data between the 10th and 90th percentile are shown as a rectangular box, divided at the median (black line), while the lower and upper portions are indicated by lines drawn to the extreme values of the range. More information about data distribution are provided adding mean value of each variable (red dots). Points outside the portions can be considered outliers. (**A**) Boxplot for distribution of lymphocytes B subpopulations divided by blood type. (**B**) Boxplot for distribution of lymphocytes T-CD4+ subpopulations divided by blood type. (**C**) Boxplot for distribution of lymphocytes T-CD8+ subpopulations divided by blood type.

The distribution of each of the selected lymphocyte subpopulation stratified by blood type is represented in the Appendix (Figure A1A–C).

## 4. Discussion

Since being declared a pandemic in March 2020, COVID-19 disease, mediated by the SARSCoV-2 infection, has represented a global public health emergency provoking billions of deaths around the world. [18]. In particular, COVID-19 disease presents a large spectrum of symptoms, ranging from asymptomatic to critical complications, such as multi-organ failure manifestation [19]. With a greater incidence in males than females and a higher incidence between the ages of 45 and 60, this has caused a collapse of public health with serious economic and psychological repercussions [20]. The immune memory components present considerable heterogeneity; therefore, it is essential to understand the role of immunity linked to the CD4+ or CD8+ phenotype in T lymphocytes, that can avoid a severe evolution of the disease. From the pandemic context, it has emerged that susceptibility to reinfection, despite previous infection and/or administration of repeated vaccine doses over time, is a serious issue today and there is still much to study, characterize and investigate.

What is certain is that SARS-CoV-2 is a virus with a short memory. Today, SARS-CoV-2 already represents an opponent to defeat, and the immune adaptive system seems to be crucial for the immune response to SARS-CoV-2 infection. Several studies highlight the correlation of COVID-19 severity and/or SARS-CoV-2 infection susceptibility to the immune-memory players. Peng et al. [21] demonstrated that the cytotoxic TCD8+ lymphocytes specific for SARS-CoV-2 are important to improve the elimination of the infected cells. In 2022, Beserra et al. [22] correlated the high expression of programmed death protein 1 (PD-1) and sPD-L1 levels in TCD4+ and CD19+ to COVID-19 severity, promoting PD-1/PDL-1 axis as a severity-associated biomarker in SARS-CoV-2-infected patients. Furthermore, Srivastava R. et al. [23] showed very interesting data about the phenotypically and functionally senescent and exhausted CD56+CD57+PD-1+ NK correlated to the severity disease in unvaccinated COVID-19 patients. They also detected a senescent T-cell effector memory phenotype in COVID-19 SYMP individuals (CD57+CD8+ TEM and CD57+CD8+ TEMRA cells). In particular, Agarwal J. et al. [24] showed the useful monitoring of T-cell populations and the CD8 subset to differentiate the COVID-19 patients’ severity, highlighting the increase in the CD8 subsets Tc Naïve-Tim3+, Tc EM-Tim-3+ and Tc CM-Tim-3+ in severe patients compared to mild patients [24]. In further work in 2020, De Biasi S. et al. [25] found that compared with healthy controls, COVID-19 patients showed several alterations in T-cell naïve, central memory, effector memory, regulatory T cells and PD1+CD57+ exhausted pattern cells. On the other hand, altered B cell subsets in acute COVID-19 disease was recorded in the convalescence phase by Shuwa A. et al. [26], with a circulating plasma blast frequency positively associated with IgA/IgG and negatively with IgM. These data promote an expansion of class-switched antibodies in COVID-19 patients.

Failure in B-cell activation or B-cell dysfunction can lead to a severe form of the disease and/or a non-performant response upon vaccination. Immunodeficiency subjects with low level of production of cytokine IL-6 showed a better disease outcome; hence, the evidence highlights a controversial role of B cells in disease progression [27]. These, together with other literature, highlight the need to improve the phenotypical lymphocyte subset characterization related to COVID-19 disease. Unique immunoregulatory system mediated by T-cell exhaustion and suppressive cytokines such as IL-10 are responsible for limiting excessive inflammation and play an important role in homeostasis in the lungs. A balance in the levels of immunoactivation and immunosuppression may therefore be crucial in the host defense against highly pathogenic coronavirus infection. Intriguingly, B-cell production of IL-10 was higher in convalescent patients with good clinical outcomes compared to patients with poor outcomes. An increase in IL-10+ B cells was associated with positive long-term outcomes and the resolution of lung pathology in COVID-19 patients may occur by suppressing excess inflammation activity [26]. This approach allowed us to assess the impact of COVID-19 hospitalization on any subsequent immune response, as opposed to the development of SARS-CoV2-specific memory; in fact IL-10+ CD4+ T cells were expanded in acute COVID-19 patients, but this was not observed in convalescent patients [26].

An important field of investigation about the susceptibility to SARS-CoV-2 infection and/or the severity of disease concerns the stratification by blood groups membership. This correlation arises from already well-known studies about the susceptibility to other viruses’ infection based on blood type, such as Norwalk virus and Hepatitis B [28,29]. Finally, Zhao J. et al. [30], demonstrated the predisposition of blood group A to the risk of infection, whereas group 0 was associated with a minor susceptibility in a cohort of Chinese COVID-19 patients; differences among the immune profile of ABO blood types were detected on lymphocyte T subpopulations. In particular, the CD3 subpopulation resulted in being lower in A blood type than in O blood type. In the same way, CD8 subpopulation percentage was lower in A and B blood type with respect to O blood type. CD4 gated on CD3 percentage was higher in B than in O blood type. On the contrary, CD8 gated on the CD3 subpopulation resulted higher in O blood type than in B blood type.

Together, these findings can help explain the varying global distribution of the virus, as well novel strategies of prevention or intervention [7,14,46,47,48,49,50]. They also enable the identification of individuals at higher risk of infection or poorer outcomes [19,49,51,52,53,54]. Additionally, by identifying the ABO blood type that binds viral peptides with greater affinity, these results can assist in developing population-specific vaccination strategies.

Future research is necessary to validate these findings in different patient populations and to investigate whether other variables, such as HLA loci, contribute to the susceptibility to SARS-CoV-2 infection and the progression of COVID-19.

## 5. Conclusions

Data in several studies show a correlation between ABO blood type and the severity of the disease, or with the patient’s outcome and/or susceptibility to infection. In our study, we highlight the importance of the immune pattern in relation to blood groups. The interpretation of the data may be important to characterize the susceptibility to infections in subjects belonging to different blood groups, from a lymphocyte phenotyping point of view. Observing variations in effector lymphocyte or memory markers could highlight the difference that leads to a different susceptibility to infection based on blood groups. Here, we characterize a T-cell subset profile able to understand the change inside the memory population, to discriminate the vaccine-mediated memory efficacy in respect to the virus direct exposition. We observed that O blood type CD3, CD8 and CD8 gated on CD3 subpopulation has a higher percentage, whereas CD4 gated on CD3 has lower percentage, with respect to other blood type. This profile may explain previously reported differences, but this research needs further investigation.

## Figures and Tables

**Table 1:** Lymphocytes B (A) and T (B) subpopulation panel investigated.

**Lymphocytes B (%)**	
CD19	
NAIVE B CELLS: IgD+ CD27− GATED ON CD19	
MARGINAL ZONE B CELLS: IgD+ CD27+ GATED ON CD19	
B CELLS IgM+ IgD+ GATED ON CD19+	
CD27+ CD38− GATED ON IgM+ IgD+ (UNSWITCHED MEMORY B CELLS)	
CD38- CD27+ GATED ON IgM− IgD− (SWITCHED MEMORY B CELLS)	
CD38high CD27+ GATED ON IgD− IgM− (PLASMABLAST)	
A	
**Lymphocytes T CD4+ (%)**	**Lymphocytes T CD8+ (%)**
CD3	CD3
CD4 (HELPER)	CD8 (CYTOTOXIC)
CD4 GATED ON CD3	CD8 GATED ON CD3
CD4+ CD57+	CD8+ CD57+
CD4+ CD27+ CD28+	CD8+ CD27+ CD28+
PD1+ CD4+	PD1+ CD8+
NAIVE T CELLS CD197+ CD45Ra+	NAIVE T CELLS CD197+ CD45Ra+
EFFECTOR T CELLS CD197− CD45Ra+	EFFECTOR T CELLS CD197− CD45Ra+
EFFECTOR MEMORY T CELLS CD197− CD45Ra-	EFFECTOR MEMORY T CELLS CD197− CD45Ra−
CENTRAL MEMORY T CELLS CD197+(CCR7) CD45Ra−	CENTRAL MEMORY T CELLS CD197+(CCR7) CD45Ra−
B	

**Table 2:** General characteristics of the enrolled population.

Enrolled Population Variable	Study Population (N = 72)
***Age***	
Median (IQR)	46.0 (20.0–64.0)
***Sex***	
F	23 (31.9%)
M	49 (68.1%)
***Province of residence***	
Benevento	2 (2.8%)
Caserta	64 (88.9%)
Napoli	5 (6.9%)
Roma	1 (1.4%)
***Blood type***	
A	21 (29.2%)
B	22 (30.6%)
0	29 (40.3%)
***Rhesus factor***	
−	6 (8.3%)
+	66 (91.7%)
***Previous SARS-CoV-2 infection***	
Yes	51 (70.8%)
No	21 (29.2%)
***Number of SARS-CoV-2 infection***	
0	22 (30.6%)
1	47 (65.3%)
2	3 (4.2%)
***Doses of SARS-CoV-2 vaccine received***	
1	2 (2.8%)
2	10 (13.9%)
3	58 (80.6%)
4	2 (4.8%)

**Table 3:** Descriptive analysis of general characteristic of enrolled population and white blood cell values stratified by blood type.

Variables	Blood Type			*p*-Value
	A	B	0	
	(N = 21)	(N = 22)	(N = 29)	
***Age***				0.355
Mean (SD)	44.9 (11.4)	47.3 (11.6)	42.6 (11.9)	
***Sex***				0.503
F	8 (38.1%)	8 (36.4%)	7 (24.1%)	
M	13 (61.9%)	14 (63.6%)	22 (75.9%)	
***City of residence***				0.127
Benevento	0 (0%)	0 (0%)	2 (6.9%)	
Caserta	20 (95.2%)	21 (95.5%)	23 (79.3%)	
Napoli	0 (0%)	1 (4.5%)	4 (13.8%)	
Roma	1 (4.8%)	0 (0%)	0 (0%)	
***Previous SARS-CoV-2 infection***				0.340
Yes	17 (81.0%)	16 (72.7%)	18 (72.7%)	
*No*	4 (19.0%)	6 (27.3%)	11 (27.3%)	
***Number of SARS-CoV-2 infection***				0.390
0	4 (19.0%)	6 (27.3%)	11 (37.9%)	
1	15 (71.4%)	16 (72.7%)	17 (58.6%)	
2	2 (9.5%)	0 (0%)	1 (3.4%)	
***Doses of SARS-CoV-2 vaccine received***				0.083
1	1 (4.8%)	1 (4.5%)	0 (0%)	
2	6 (28.6%)	1 (4.5%)	3 (10.3%)	
3	14 (66.7%)	20 (90.9%)	24 (82.8%)	
4	0 (0%)	0 (0%)	2 (6.9%)	
**White blood cells**				
***Lymphocytes %***				0.212
Mean (SD)	34.2 (8.4)	32.9 (8.92)	36.7 (6.9)	
***Lymphocytes abs. count***				0.472
Mean (SD)	1.9 (0.67)	2.0 (0.6)	2.1 (0.6)	
***Monocytes %***				0.761
Mean (SD)	6.3 (1.7)	6.5 (1.3)	6.7 (1.7)	
***Monocytes abs. count***				0.284
Mean (SD)	0.7 (1.4)	0.4 (0.1)	0.4 (0.2)	
Granulocytes %				0.593
Mean (SD)	58.1 (9.2)	58.6 (8.3)	56.4 (6.9)	
***Granulocytes abs. count***				0.785
Mean (SD)	3.5 (1.4)	3.7 (1.2)	3.4 (1.3)	

**Table 4:** Descriptive analysis of lymphocyte B subpopulation stratified by blood type. No statistical differences were detected.

Lymphocytes B Subpopulations (%)	Blood Type			*p*-Value
	A	B	0	
	(N = 21)	(N = 22)	(N = 29)	
***CD19***				0.111
Median (IQR)	9.0 (7.0–11.0)	7.0 (6.0–8.8)	8.0 (6.0–9.0)	
***Naive B cells: IgD+ CD27−*** ***gated on CD19***				0.437
Median (IQR)	55.0 (45.0–65.0)	63.0 (57.0–68.8)	64.0 (48.0–71.0)	
***Marginal Zone B cells: IgD+ CD27+ gated on CD19***				0.497
Median (IQR)	9.0 (4.0–13.0)	6.0 (3.3–8.0)	6.0 (4.0–12.0)	
***B cells IgM+ IgD+ gated on CD19+***				0.113
Mean (SD)	42.2 (19.4)	37.2 (20.4)	47.6 (13.0)	
***CD27+ CD38− gated on IgM+ IgD+ (unswitched memory B cells)***				0.939
Mean (SD)	18.0 (8.0–20.0)	16.0 (9.0–24.8)	17.0 (9.0–25.0)	
***CD38- CD27+ GATED ON IgM*** ***−*** ***IgD*** ***−*** ***(switched memory B cells)***				0.284
Mean (SD)	59.1 (14.6)	50.5 (20.2)	56.7 (19.7)	
***CD38high CD27+ gated on IgD*** ***−*** ***IgM*** ***−*** ***(plasmablast)***				0.522
Mean (SD)	2.0 (1.0–6.0)	1.5 (0.0–3.0)	2.0 (0.0–4.0)	

**Table 5:** Descriptive analysis of lymphocytes T-CD4 and T-CD8 subpopulations stratified by blood type. CD3, CD4 gated on CD3, CD8 (cytotoxic) and CD8 gated on CD3 resulted in being statistically different. Pairwise *t*-test or non-parametric Mann-Whitney U test with Benjamini–Hochberg correction for multiple testing led to a significant result for the comparisons A versus O blood type for CD3 and CD8 (*p* = 0.043, *p* = 0.002), whereas A versus O blood type for CD4 gated on CD3 CD8 and CD8 gated on CD3 (*p* = 0.047, *p* = 0.008, *p* = 0.031). *: A *p*-value < 0.05 was considered statistically significant.

Lymphocytes T Subpopulations (%)	Blood Type			*p*-Value *
	A	B	0	
	(N = 21)	(N = 22)	(N = 29)	
***CD3***				**0.026**
Mean (SD)	72.5 (8.94)	73.1 (8.44)	77.7 (4.93)	
***CD4 (helper)***				0.219
Mean (SD)	51.9 (10.0)	51.9 (9.3)	47.8 (9.6)	
***CD4 gated on CD3***				**0.044**
Median (IQR)	68.0 (57.0–72.0)	69.5 (60.0–74.0)	60.0 (54.0–66.0)	
***CD4+ CD57+***				0.679
Median (IQR)	1.0 (0.4–5.0)	2.0 (0.9–5.0)	2.0 (0.4–4.0)	
***CD4+ CD27+ CD28+***				0.961
Median (IQR)	88.0 (81.0–90.0)	88.5 (73.8–91.0)	87.0 (81.0–92.0)	
***PD1+ CD4+***				0.090
Median (IQR)	19.0 (13.0–23.0)	13.0 (11.0–18.8)	13.0 (9.0–20.0)	
***Naive T cells CD197+ CD45Ra+ CD4+***				0.611
Mean (IQR)	38.4 (11.5)	36.8 (17.6)	41.1 (16.8)	
***Effector T cells CD197*** **−** ***CD45Ra+ CD4+***				0.061
Median (IQR)	1.0 (0.4–3.0)	2.5 (1.0–4.5)	0.2 (0.0–3.0)	
***Effector memory T cells CD197− CD45Ra-CD4+***				0.240
Median (IQR)	13.0 (8.0–17.0)	8.0 (6.3–18.0)	8.0 (5.0–12.0)	
***Central memory T cells CD197+ (CCR7) CD45Ra- CD4+***				0.980
Median (IQR)	47.0 (38.0–52.0)	45.5 (38.8–51.8)	45.0 (38.0–53.0)	
***CD8 (cytotoxic)***				**<0.001**
Mean (SD)	19.3 (7.5)	20.5 (9.8)	29.0 (10.8)	
***CD8 gated on CD3***				**0.029**
Median (IQR)	26.0 (20.0–35.0)	26.5 (18.3–31.0)	32.0 (28.0–39.0)	
***CD8+ CD57+***				0.541
Median (IQR)	22.0 (11.0–38.0)	26.5 (17.0–45.5)	26.0 (18.0–38.0)	
***CD8+ CD27+ CD28+***				0.769
Mean (SD)	55.2 (18.3)	51.2 (18.5)	52.9 (17.4)	
***PD1+ CD8+***				0.080
Mean (SD)	30.9 (14.7)	22.7 (14.5)	30.3 (11.3)	
***Naive T cells CD197+ CD45Ra+ CD8+***				0.483
Median (IQR)	31.0 (28.0–41.0)	26.0 (10.5–45.5)	30.0 (20.0–50.0)	
*Effector T cells CD197* **−** *CD45Ra+ CD8+*				0.783
Median (IQR)	25.0 (13.0–40.0)	28.0 (19.3–41.5)	28.0 (17.0–46.0)	
*Effector memory T cells CD197− CD45Ra− CD8+*				0.294
Median (IQR)	18.0 (15.0–27.0)	18.5 (13.0–24.0)	16.0 (11.0–20.0)	
*Central memory T cells CD197+ (CCR7) CD45Ra− CD8+*				0.177
Median (IQR)	13.0 (10.0–16.0)	12.0 (10.0–19.8)	9.0 (7.0–12.0)	

## Data Availability

The original contributions presented in the study are included in the articlefurther inquiries can be directed to the corresponding author.

## Appendix A

**Table A1:** Distribution of Rhesus factor among enrolled population expressed as absolute and relative percentage frequencies.

	Blood Type			Study Population (N = 72)
	A	B	0	
	(N = 21)	(N = 22)	(N = 29)	
**Rhesus factor**				
−	3 (14.3%)	2 (9.1%)	1 (3.4%)	6 (8.3%)
+	18 (85.7%)	20 (90.9%)	28 (96.6%)	66 (91.7%)
